# Supplementary material for: RCX—an R package adapting the Cytoscape Exchange format for biological networks
Source: Bioinform Adv. 2022 Mar 31;2(1):vbac020. doi: 10.1093/bioadv/vbac020 (PMC9710607; doi:10.1093/bioadv/vbac020)
Supplement: vbac020_Supplementary_Data [file vbac020_supplementary_data.zip › Appendix - The RCX and CX Data Model.pdf]

Appendix: The RCX and CX Data Model

Florian J. Auer[1*]

[1]University Augsburg

[*]Florian.Auer@informatik.uni-augsburg.de

2021-10-26

Contents

1 CX data structure
- 1.1 Aspect dependencies

2 Data types

3 NDEx conventions
- 3.1 Handling of Identifiers
- 3.2 Citations

4 Meta aspects
- 4.1 metaData
- 4.2 status

5 Core aspects
- 5.1 nodes
- 5.2 edges
- 5.3 nodeAttributes
  - 5.3.1 NDEx conventions
- 5.4 edgeAttributes
- 5.5 networkAttributes
  - 5.5.1 NDEx conventions
- 5.6 cartesianLayout

6 Cytoscape aspects
- 6.1 cyGroups
- 6.2 CyVisualProperties
  - 6.2.1 CyVisualProperties (CX)
  - 6.2.2 CyVisualProperties (RCX)
- 6.3 cyHiddenAttributes
- 6.4 cyNetworkRelations
- 6.5 cySubNetworks
- 6.6 cyTableColum

7 Deprecated aspects

8 Session info

1 CX data structure

The CX data structure can be divided into three main classes:

- Meta aspects: necessary for data transmission
  - metaData
  - status
- Core aspects: essential for defining a network
  - nodes
  - edges
  - nodeAttributes
  - edgeAttributes
  - networkAttributes
  - cartesianLayout
- Cytoscape aspects: aspects needed to define the visual layout of a network in Cytoscape
  - cyGroups
  - cyVisualProperties
  - cyHiddenAttributes
  - cyNetworkRelations
  - cySubnetworks
  - cyTableColumn

1.1 Aspect dependencies

Aspects with IDs:

- nodes
- edges
- cyGroups
- cySubnetworks

Aspects, that reference IDs:

- edges reference nodes by “source” and “target”
- nodeAttributes reference nodes by “propertyOf”
- edgeAttributes reference edges by “propertyOf”
- cartesianLayout references cySubnetworks by “view”
- cyGroups reference nodes by “nodes” and edges by “internalEdges” and “externalEdges”
- sub-aspect cyVisualProperty of cyVisualProperties references cySubnetworks by “appliesTo” and “view”
- cySubnetworks reference nodes by “nodes” and edges by “edges”

2 Data types

CX is a JSON based format, that means that JavaScript data types are used. Those will be mapped to and from R data types as follows:

data type	example	R data type
boolean	“true”	logical
double	“2.3”	numeric
integer	“23”	integer
long	“123456”	integer
string	“blue”	character
list_of_boolean	[“true”,“false”]	list(logical)
list_of_double	[“2.3”,“4.5”]	list(numeric)
list_of_integer	[“23”,“45”]	list(integer)
list_of_long	[“23”,“45”]	list(integer)
list_of_string	[“Marco”,“Polo”]	list(character)

3 NDEx conventions

3.1 Handling of Identifiers

All identifiers should be either:

- <prefix>:<id> format
- a string without " : "
- a URI

3.2 Citations

It is recomended to use the attribute “citation” on edges or nodes to store citations to specify literature references or other sources of information that are relevant to the network. Citations are primarily described by five dublin core terms. The “dc” prefix is implicitly interpreted as referencing dublin core in the context of the citations aspect.

The five primary dublin core terms defined for citations are:

- dc:title
  - http://dublincore.org/documents/2012/06/14/dcmi-terms/?v=terms#terms-title
- dc:contributor
  - http://dublincore.org/documents/2012/06/14/dcmi-terms/?v=terms#terms-contributor
- dc:identifier
  - http://dublincore.org/documents/2012/06/14/dcmi-terms/?v=terms#terms-identifier
  - Ideally citations make use of the “identifier” key to link to their source. This can be a URI, but might also use the “source:id” format, where source is usually “pmid” or “doi”.
- dc:type
  - http://dublincore.org/documents/2012/06/14/dcmi-terms/?v=terms#terms-type
- dc:description
  - http://dublincore.org/documents/2012/06/14/dcmi-terms/?v=elements#description
  - This should be a description of the resource, and it is preferable that information that can be expressed in more specific attributes should not be in this attribute. The “description” attribute could contain the title, authors, and/or journal reference, but in that case it would not be expected to be machine parsable.

4 Meta aspects

4.1 metaData

As a transfer format,the owner (sender) is considered to have a complete picture of the network including all metadata and aspects associated with the network. When a sender has the choice of sending an element as either pre- or post-metadata. In RCX both are combined when converting a CX file. The content a the metaData aspect is specified as:

RCX property	RCX specifics	CX property	CX options	description
name		name	Required in pre- and post-metadata	The name of the aspect
version	default:“1.0”	version	Required in pre-metadata	version of this aspect schema (“1.1.0”, “2.0.0”, etc.)
idCounter	auto-generated	idCounter	Required if the aspect exports IDs	Integer (All Element IDs are integers; see node id, edge id, cytoscape groups)
elementCount	auto-generated	elementCount	Optional	number (integer) of elements in this aspect
consistencyGroup	default:1	consistencyGroup	Optional	An integer identifier shared by aspects to indicate that they are mutually consistent
properties		properties	Optional	An aspect-defined property list
-	*not supported*	checksum	Optional *(Deprecated)*	*NDEx CX implementation does not support this attribute. This attribute is ignored in NDEx*

4.2 status

A complete CX stream ends with a status aspect object. This aspect tells the recipient if the CX is successfully generated by the source.

Note: This aspect is auto-generated in RCX and cannot be changed!

property	options	values	description
success	Required	“true” or “false”	Was the CX successfully generated by the source
error	Required	string or ""	error message or "" if sucess==“true”

5 Core aspects

5.1 nodes

RCX property	CX property	CX options	values	description
id	@id	Required, Unique	integer	CX internal id, starts at 0, exported to metaData!
name	n	Optional	string	node name, eg. “EGFR”, “node 1”
represents	r	Optional	string	represents, eg. “HGNC:AKT1”

Note: At least one node has to be present, therefore this aspect is mandatory!

5.2 edges

RCX property	CX property	CX options	values	description
id	@id	Required, Unique	integer	CX internal id, starts at 0, exported to metaData!
source	s	Required	integer	source, reference to CX internal node id
target	t	Required	integer	target, reference to CX internal node id
interaction	i	Optional	string	intercation, eg. “binds”

5.3 nodeAttributes

RCX property	RCX specifics	CX property	options	values	description
propertyOf		po	Required	integer	property of, reference to CX internal node id
name		n	Required	string	attribute name, eg. “weight”, “name”, “selected”
value		v	Required	string or list of strings	attribute value(s), eg. [“2”, “0.34”, “2.3”], “Node 6”, “true”

RCX property	RCX specifics	CX property	options	values	description
dataType	default:“string”	d	Optional	string	data type, default “string”
isList	default:FALSE	d	Optional	string	If set to TRUE, the CX data type will be modified
subnetworkId	default:NA	s	Optional	integer	reference to CX internal subnetwork id

5.3.1 NDEx conventions

names	description
alias	alternative identifiers for the node. Same meaning as BioPAX “aliases”
relatedTo	identifiers denoting concepts related to the node. Same meaning as BioPAX “relatedTerms”

5.4 edgeAttributes

RCX property	RCX specifics	CX property	options	values	description
propertyOf		po	Required	integer	property of, reference to CX internal edge id
name		n	Required	string	attribute name, eg. “weight”, “name”, “selected”
value		v	Required	string or list of strings	attribute value(s), eg. [“2”, “0.34”, “2.3”], “Node 6”, “true”
dataType	default:“string”	d	Optional	string	data type, default “string”
isList	default:FALSE	d	Optional	boolean	If set to TRUE, the CX data type will be modified
subnetworkId	default:NA	s	Optional	integer	reference to CX internal subnetwork id

5.5 networkAttributes

RCX property	RCX specifics	CX property	options	values	description
name		n	Required	string	attribute name, eg. “dc:title”
value		v	Required	string or list of strings	attribute value(s), eg. "Result of heat
dataType	default:“string”	d	Optional	string	data type, default “string”
isList	default:FALSE	d	Optional	boolean	If set to TRUE, the CX data type will be modified
subnetworkId	default:NA	s	Optional	integer	reference to CX internal subnetwork id

5.5.1 NDEx conventions

names	description
name	the name of the network
description	a description of the network
version	NDEx will treat this attribute as the version of the network. Format is not controlled but best practice is to use string conforming to Semantic Versioning.
ndex:sourceFormat	used by NDEx to indicate format of an original file imported, can determine semantics as well. The NDEx UI will allow export options based on this value. Applications that alter a network such that it can no longer be exported in the format should remove the value.

5.6 cartesianLayout

Cartesian layout elements store coordinates of nodes.

RCX property	CX property	options	values	description
node	node	Required	integer	reference to CX internal node id
x	x	Required	numeric	x coordinate
y	y	Required	numeric	y coordinate
z	z	Optional	numeric	z coordinate
view	view	Optional	integer	reference to CX internal reference to CX internal subnetwork id of type view

6 Cytoscape aspects

See Cytoscape Styles on how styles are created in Cytoscape. The following aspects are based on how Cytoscape defines the visual representation of networks. For further information see Using Visual Properies in RCy3, which gives an overview of how to handle Visual properties in R automation.

6.1 cyGroups

RCX property	CX property	options	values	description
id	@id	Required, Unique	integer	CX internal id, starts at 0, exported to metaData!
n	n	Required	string	name of this group
nodes	nodes	Optional	list of integer	the nodes making up the group, reference to CX internal node id
externalEdges	external_edges	Optional	list of integer	the external edges making up the group, reference to CX internal edge id
internalEdges	internal_edges	Optional	list of integer	the internal edges making up the group, reference to CX internal edge id
collapsed	collapsed	Optional	boolean	indicate whether the group is displayed as a single node

6.2 CyVisualProperties

6.2.1 CyVisualProperties (CX)

CX property	options	values	description
properties_of	Required	string, dictionary	to indicate the element type these properties belong to, allowed values are: “network”, “nodes:default”, “edges:default”, “nodes”, “edges”
applies_to	Required	integer	the identifier of the element these properties apply to, reference to CX internal node id or edge id
view	Optional	integer	reference to CX internal subnetwork id of type view
properties	Optional	named list of strings	pairs of the actual properties, e.g. “NODE_BORDER_STROKE” : “SOLID”, “NODE_BORDER_WIDTH” : “1.5”, “NODE_FILL_COLOR” : “#FF3399”
dependencies	Required	named list of strings	pairs of the dependencies, e.g. “nodeCustomGraphicsSizeSync” : “true”, “nodeSizeLocked” : “false”
mappings	Optional	named list of named list of strings, dictionary	e.g. “NODE_BORDER_WIDTH” : {“type” : “DISCRETE”, “definition” : “COL=required,T=boolean,K=0=true,V=0=10.0”}

The JSON object model for the cyVisualProperties aspect is not suitable to be proper represented in R data structures, therefore it is split up into the main aspect and several sub-aspects. The R structure looks as follows:

```
CyVisualProperties
├──network = CyVisualProperty
├──nodes = CyVisualProperty
├──edges = CyVisualProperty
├──defaultNodes = CyVisualProperty
└──defaultEdges = CyVisualProperty

CyVisualProperty
├──properties = CyVisualPropertyProperties
│    ├──name
│    └──value
├──dependencies = CyVisualPropertyDependencies
│    ├──name
│    └──value
├──mappings = CyVisualPropertyMappings
│    ├──name
│    ├──type
│    └──definition
├──appliesTo = <reference to subnetwork id>
└──view = <reference to subnetwork id>
```

6.2.2 CyVisualProperties (RCX)

RCX property	options	values	description
network	Optional	list of CyVisualProperty	represents “property_of”=“network” of CyVisualProperties
nodes	Optional	list of CyVisualProperty	represents “property_of”=“nodes:default” of CyVisualProperties
edges	Optional	list of CyVisualProperty	represents “property_of”=“edges” of CyVisualProperties
defaultNodes	Optional	list of CyVisualProperty	represents “property_of”=“nodes:default” of CyVisualProperties
defaultEdges	Optional	list of CyVisualProperty	represents “property_of”=“edges:default” of CyVisualProperties

6.2.2.1 CyVisualProperty

RCX property	RCX specifics	options	values	description
properties	default:NA	Optional	CyVisualPropertyProperties	represents “property_of”=“network” of CyVisualProperties
depencdencies	default:NA	Optional	CyVisualPropertyDependencies	represents “property_of”=“nodes:default” of CyVisualProperties
mappings	default:NA	Optional	CyVisualPropertyMappings	represents “property_of”=“edges” of CyVisualProperties
appliesTo	default:NA	Optional	integer	reference to CX internal subnetwork id
view	default:NA	Optional	integer	reference to CX internal subnetwork id of type view

6.2.2.2 CyVisualPropertyProperties

RCX property	options	values	description
name	Required	string	name of the property, e.g. “NODE_PAINT” or “EDGE_VISIBLE”
value	Required	string	value of the property, e.g. “#FF0000” or “true”

6.2.2.3 CyVisualPropertyDependencies

RCX property	options	values	description
name	Required	string	name of the dependency, e.g. “nodeCustomGraphicsSizeSync” or “nodeSizeLocked”
value	Required	string	value of the dependency, e.g. “true” or “false”

6.2.2.4 CyVisualPropertyMappings

The JSON objects or the form `{"NODE_BORDER_WIDTH" : {"type" : "DISCRETE", "definition" : "COL=required,T=boolean,K=0=true,V=0=10.0"}}` are split up as follows:

RCX property	options	values	description
name	Required	string	name of the property, e.g. “NODE_BORDER_WIDTH”
type	Required	string	value of the property, e.g. “DISCRETE”
definition	Required	string	value of the property, e.g. “COL=required,T=boolean,K=0=true,V=0=10.0”

6.3 cyHiddenAttributes

RCX property	RCX specifics	CX property	options	values	description
name		n	Required	string	name of this hidden attribute
value		v	Required	string or list of strings	attribute value(s), eg. “layoutAlgorithm”
dataType	default:“string”	d	Optional	string	data type, default “string”
isList	default:FALSE	d	Optional	boolean	If set to TRUE, the CX data type will be modified
subnetworkId	default:NA	s	Optional	integer	reference to CX internal subnetwork id

6.4 cyNetworkRelations

RCX property	RCX specifics	CX property	options	values	description
child		c	Required	integer	child network, reference to CX internal subnetwork id
parent	default:NA	p	Optional	integer	parent network, reference to CX internal subnetwork id
name		name	Optional	string	the name of the child network; if missing, default is reader-dependent
isView	default:FALSE	r	Optional	boolean	relationship type, default “subnetwork”

6.5 cySubNetworks

RCX property	CX property	options	values	description
id	@id	Required, Unique	integer	CX internal id, starts at 0, exported to metaData!
nodes	nodes	Optional	list of integer or “all”	the nodes making up the group, reference to CX internal node id
edges	edges	Optional	list of integer or “all”	the edges making up the group, reference to CX internal edge id

6.6 cyTableColum

These elements are used to represent Cytoscape table column labels and types. Its main use is to disambiguate empty table columns.

RCX property	RCX specifics	CX property	options	values	description
name		n	Required	string	name of the column; usually the different attributes of nodes, edges and networks

RCX property	RCX specifics	CX property	options	values	description
appliesTo	“nodes”, “edges”, or “networks”	applies_to	Required	string: “node_table”, “edge_table”, or “network_table”	indicates the Cytoscape table this applies to
dataType	default:“string”	d	Optional	string	data type, default “string”
isList	default:FALSE	d	Optional	boolean	If set to TRUE, the CX data type will be modified
subnetworkId	default:NA	s	Optional	integer	reference to CX internal subnetwork id

Note: Cytoscape does not currently support table columns for the root network, but this is option is included here for consistency

7 Deprecated aspects

property	description
ndexStatus	NDEx server will no longer generate this aspect from the server side. Network last updated before the v2.4.0 release will still have this aspect. Users can use the “Get Network Summary” API function to get the status and other information of a network.
citations	We recommend using the attribute “citation” on edges or nodes to store citations.
nodeCitations	We recommend using the attribute “citation” on nodes to store citations.
edgeCitations	We recommend using the attribute “citation” on edges to store citations.
Supports	We recommend using the attribute “supports” on edges or nodes.
nodeSupports	We recommend using the attribute “supports” on nodes.
edgeSupports	We recommend using the attribute “supports” on edges.
functionTerms	No recommendations for alternative representation.
reiefiedEdges	No recommendations for alternative representation.
@context	We recommend using the attribute “@context” on networks. The value of this attribute is a serialized string from a JSON dictionary that has a prefix as its key and a URL as its value. @context maps terms to IRIs. Terms are case sensitive.
provenanceHistory	The server will just treat this as an opaque aspect. For recording the origin and significant events of this network, we recommend to use network attributes to store them. We recommend using prov:wasGeneratedBy and prov:wasDerivedFrom for the event and the list of contributing network URLs.

Note: Starting from v2.4.0, NDEx server will no longer generate these aspects from the server side. Networks last updated before the v2.4.0 release will still have these aspects.

8 Session info

```
sessionInfo()
```

```
## R version 4.0.3 (2020-10-10)
## Platform: x86_64-pc-linux-gnu (64-bit)
## Running under: Ubuntu 20.04.3 LTS
##
## Matrix products: default
## BLAS:   /usr/lib/x86_64-linux-gnu/blas/libblas.so.3.9.0
## LAPACK: /usr/lib/x86_64-linux-gnu/lapack/liblapack.so.3.9.0
##
## locale:
##  [1] LC_CTYPE=en_US.UTF-8       LC_NUMERIC=C
##  [3] LC_TIME=de_DE.UTF-8        LC_COLLATE=en_US.UTF-8
##  [5] LC_MONETARY=de_DE.UTF-8    LC_MESSAGES=en_US.UTF-8
##  [7] LC_PAPER=de_DE.UTF-8       LC_NAME=C
##  [9] LC_ADDRESS=C               LC_TELEPHONE=C
## [11] LC_MEASUREMENT=de_DE.UTF-8 LC_IDENTIFICATION=C
##
## attached base packages:
## [1] stats     graphics  grDevices utils     datasets  methods   base
##
## other attached packages:
## [1] BiocStyle_2.18.1
##
## loaded via a namespace (and not attached):
##  [1] bookdown_0.24       digest_0.6.28       magrittr_2.0.1
##  [4] evaluate_0.14       rlang_0.4.12        stringi_1.7.5
##  [7] jquerylib_0.1.4     rmarkdown_2.11      tools_4.0.3
## [10] stringr_1.4.0       xfun_0.27           yaml_2.2.1
## [13] fastmap_1.1.0       compiler_4.0.3      BiocManager_1.30.16
## [16] htmltools_0.5.2     knitr_1.36
```
