# Supplementary figures and images for: RCX—an R package adapting the Cytoscape Exchange format for biological networks
Source: Bioinform Adv. 2022 Mar 31;2(1):vbac020. doi: 10.1093/bioadv/vbac020 (PMC9710607; doi:10.1093/bioadv/vbac020)

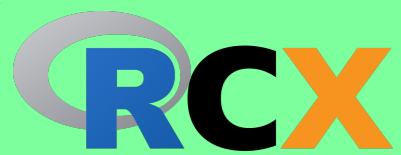

# CHEAT SHEET

## DATA MODEL STRUCTURE

RCX

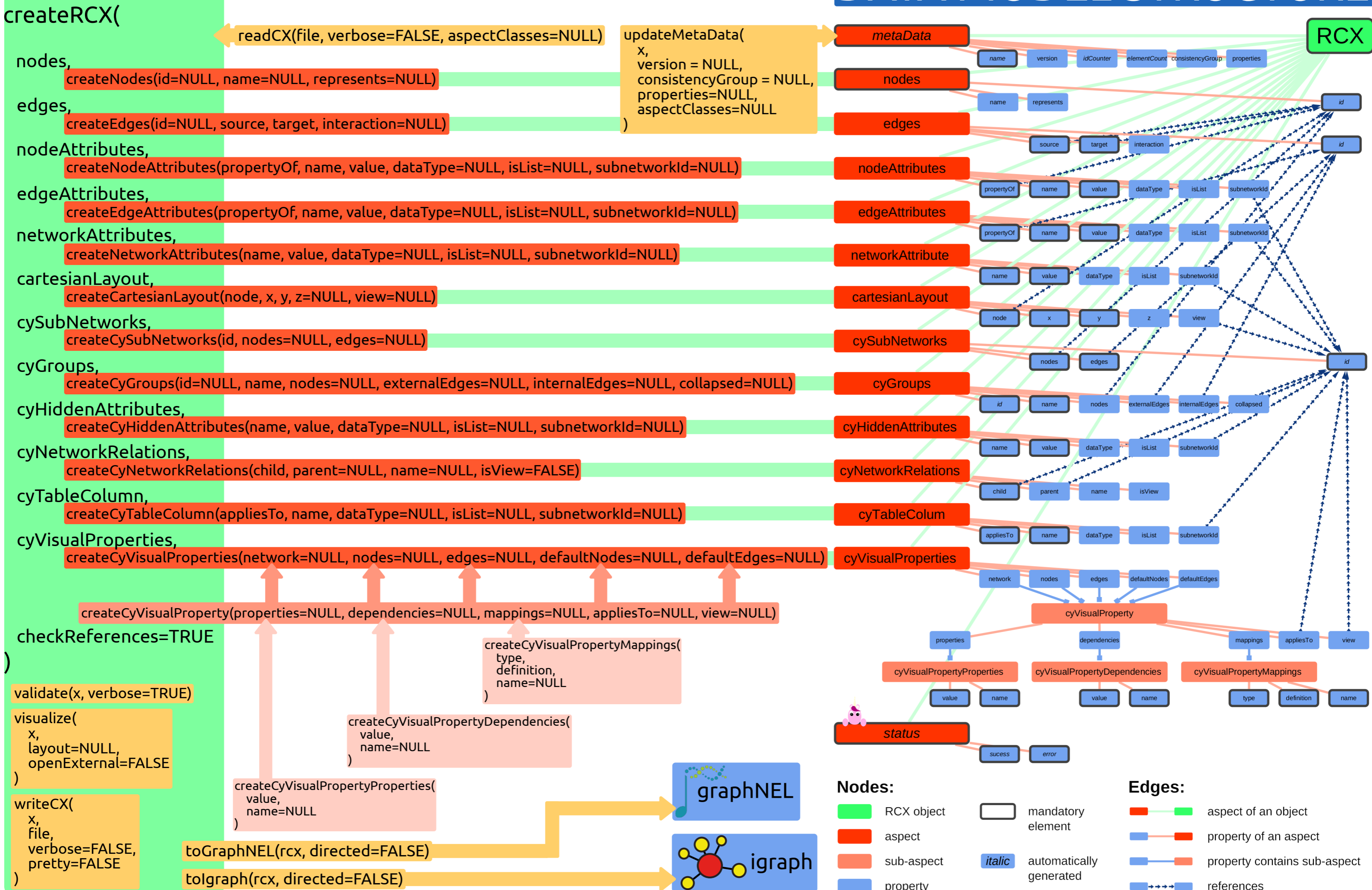

Supplement: vbac020_Supplementary_Data [file vbac020_supplementary_data.zip › RCX Cheat Sheet.pdf]
